# Supplementary figures and images for: Validation of Plasmodium falciparum deoxyhypusine synthase as an antimalarial target
Source: PeerJ. 2019 Apr 17;7:e6713. doi: 10.7717/peerj.6713 (PMC6475138; doi:10.7717/peerj.6713)

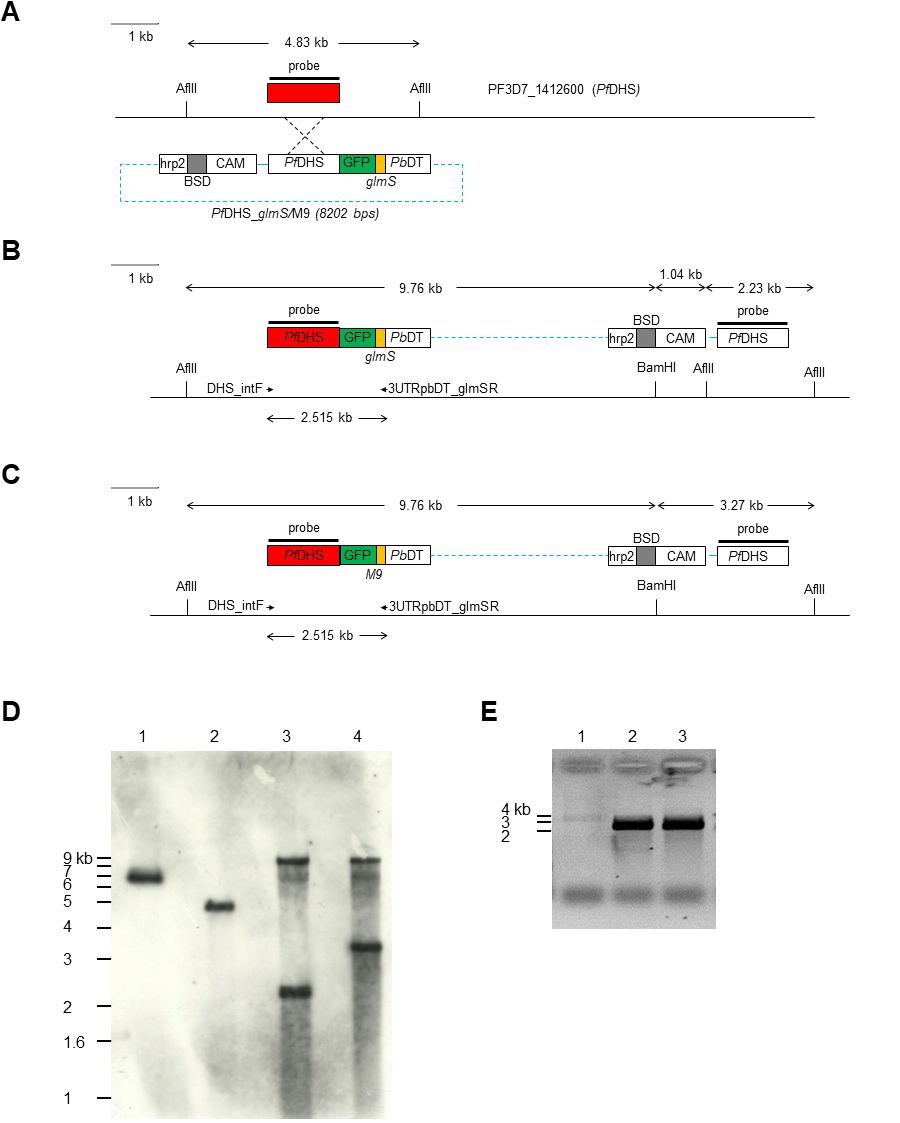

Supplement: Supplemental Information 1 — (A) Schematic diagram of the Plasmodium falciparum 3D7 genomic region encompassing the PF3D7_1412600 (PfDHS) gene, drawn to scale. The PfDHS annotated gene model exon is indicated by a red box. The locations of AflII restriction sites and the Southern blot probe hybridizing region are indicated on the genomic DNA line. The transfection plasmids PfDHS_glmS and PfDHS_M9 integrated into the PfDHS locus in transgenic parasites via single-crossover recombination, as indicated by the dashed lines. Functional elements in transfection plasmids are depicted as boxes: the blasticidin-S-deaminase (BSD) selection marker gene, camodulin gene promoter (CAM), histidine-rich protein two gene terminator (hrp2), PfDHS homologous targeting sequence (PfDHS), GFP gene (GFP), glmS riboswitch (wild-type or M9 variant; glmS), and P. berghei dihydrofolate reductase-thymidylate synthase gene terminator (PbDT). Plasmid backbone (not to scale) is depicted as a dashed blue line. (B) Schematic diagram of the PfDHS gene region in transgenic parasites with integrated PfDHS_glmS plasmid (all elements drawn to scale). The locations of BamHI and AflII restriction sites and the Southern blot probe hybridizing regions are indicated on the genomic DNA line. The locations of DHS_intF and 3UTRpbDT_glmSR primer binding sites for integration-specific PCR assay are indicated by arrows. (C) Schematic diagram of the PfDHS gene region in transgenic parasites with integrated PfDHS_M9 plasmid (all elements drawn to scale). The locations of BamHI and AflII restriction sites and the Southern blot probe hybridizing regions are indicated on the genomic DNA line. The locations of DHS_intF and 3UTRpbDT_glmSR primer binding sites for integration-specific PCR assay are indicated by arrows. (D) Southern blot of P. falciparum genomic DNA. DNA samples were digested with BamHI and AflII, separated by agarose electrophoresis and blotted onto a Hybond N+ nylon membrane (GE healthcare). The membrane was hybridized with a DIG-la [file peerj-07-6713-s001.png]

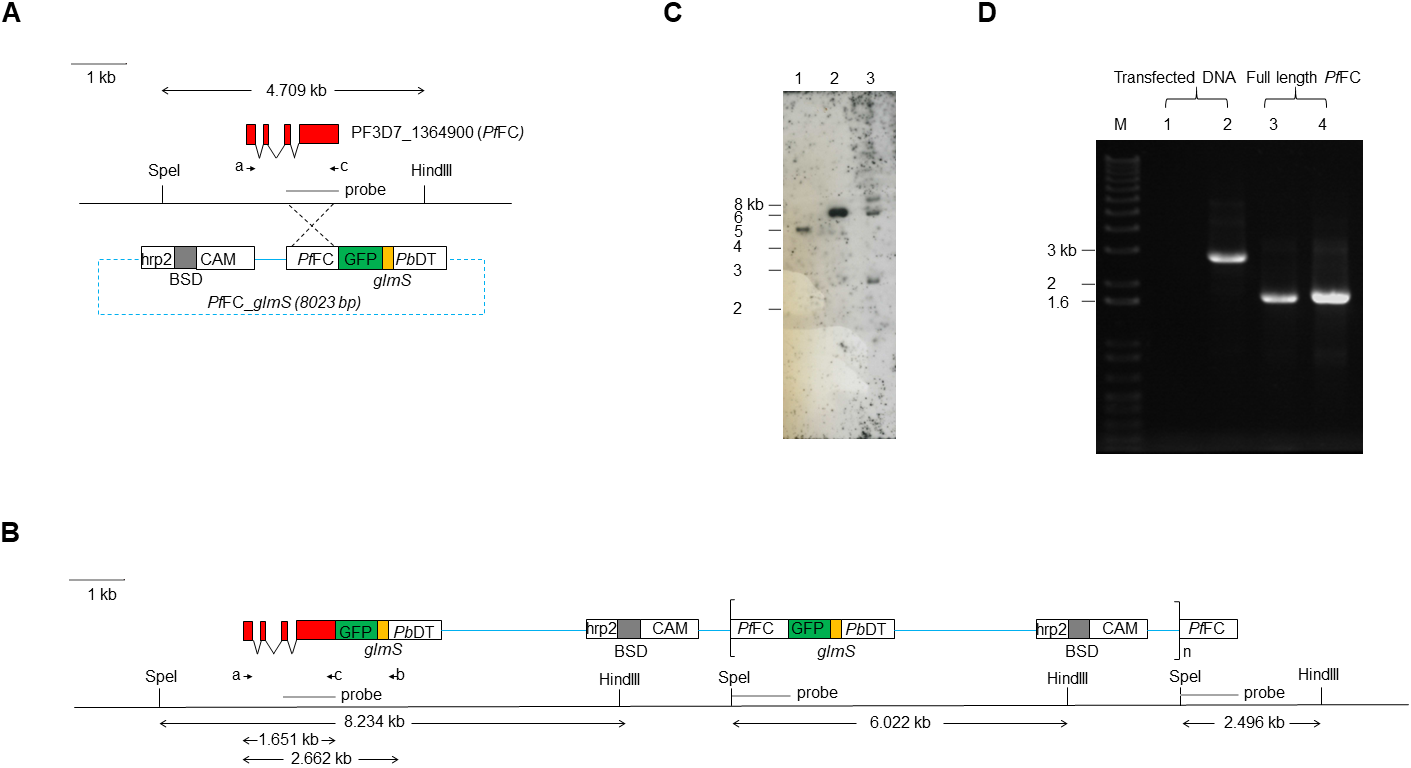

Supplement: Supplemental Information 2 — (A) Schematic diagram of the Plasmodium falciparum 3D7 genomic region encompassing the PF3D7_1364900 (PfFC) gene, drawn to scale. The PfFC annotated gene model exons are indicated by red boxes. The PfFC_glmS transfection plasmid integrated into the PfFC locus in transgenic parasites via single-crossover recombination, indicated by the dashed lines. Functional elements in transfection plasmids are depicted as boxes: the blasticidin-S-deaminase (BSD) selection marker gene, camodulin gene promoter (CAM), histidine-rich protein two gene terminator (hrp2), PfFC homologous targeting sequence (PfFC), GFP gene (GFP), glmS riboswitch (glmS), and P. berghei dihydrofolate reductase-thymidylate synthase gene terminator (PbDT). Plasmid backbone (not to scale) is depicted as a dashed blue line. The locations of SpeI and HindIII restriction sites and the Southern blot probe hybridizing region are indicated on the genomic DNA line. (B) Schematic diagram of the PfFC gene region in transgenic parasites with integrated PfFC_glmS plasmid (all elements drawn to scale). The locations of BglIIPfFCF and 3UTRpbDT_glmSR primer binding sites for integration-specific PCR assay are indicated by arrows labeled “a” and “b,” respectively. The PfFCKpnIR control primer binding site is indicated by an arrow labeled as “c.” The locations of SpeI and HindIII restriction sites and the Southern blot probe hybridizing regions are indicated on the genomic DNA line. (C) Southern blot of P. falciparum genomic DNA. DNA samples were digested with HindIII and SpeI, separated by agarose electrophoresis and blotted onto a Hybond N+ nylon membrane (GE healthcare). The membrane was hybridized with a DIG-labeled DNA probe synthesized by PCR using primers BglIIPfFCF and PfFCKpnIR. DNA samples: lane 1, P. falciparum 3D7 parental parasite genomic DNA; lane 2, PfFC_glmS plasmid DNA; lane 3, PfFC_glmS transgenic parasite genomic DNA. The migrations of one kb+ DNA ladder (Invitrogen) bands are indicated on the left. (D) A [file peerj-07-6713-s002.png]

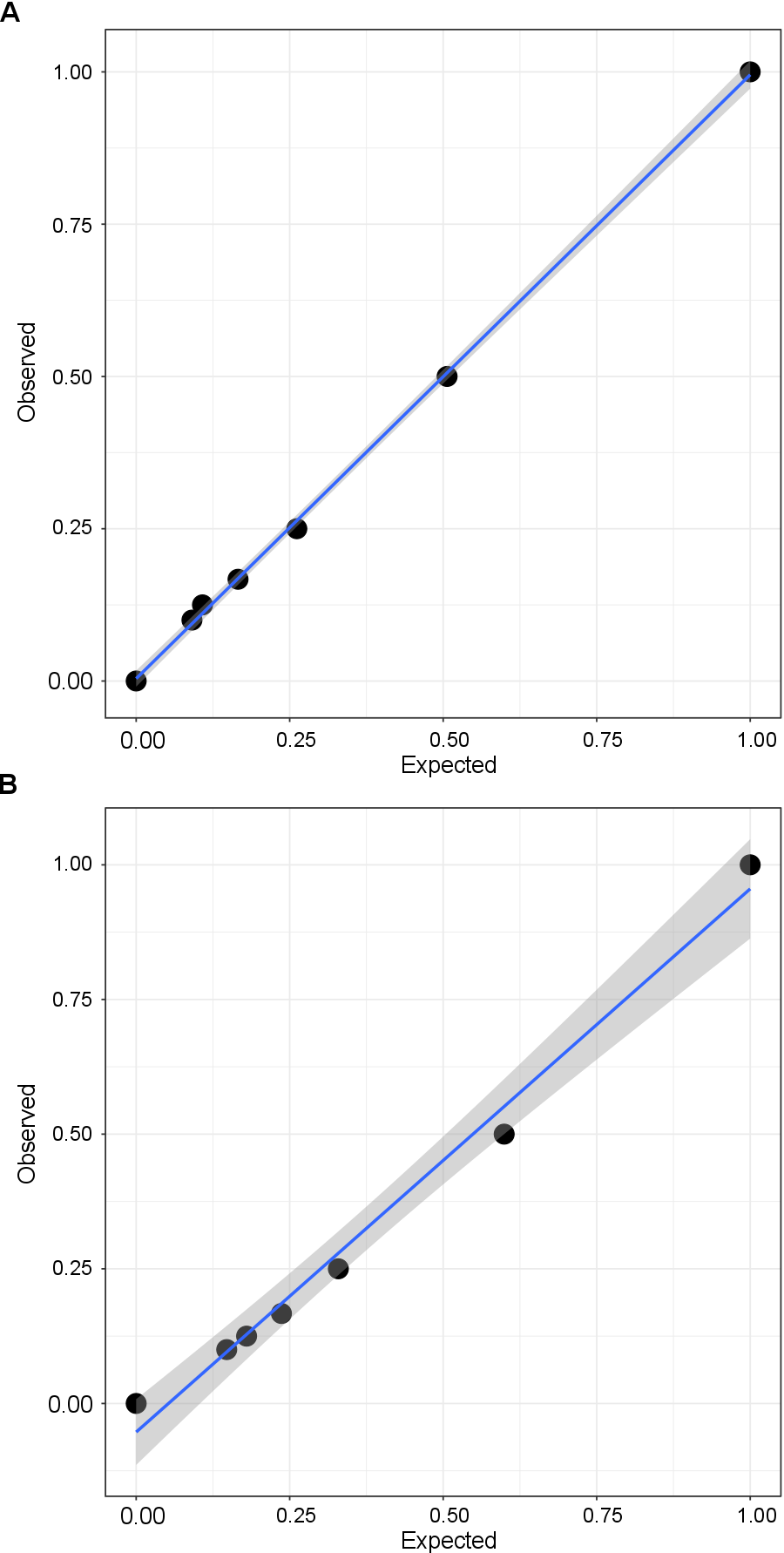

Supplement: Supplemental Information 3 — Purified genomic DNA samples from transgenic parasites were mixed in various ratios according to Nanodrop measurements of DNA concentrations. The qPCR abundances of DHS-GFP, TS-GFP, and FC-GFP amplicons were normalized to the LDH amplicon and were used to calculate DNA ratios. Pearson’s coefficient of correlation of observed ratios from qPCR data and expected ratios from known DNA concentration was calculated in R. (A) Correlation of observed and expected ratio of PfDHS_glmS and PfFC_glmS genomic DNA (Pearson’s r = 0.9996; P = 4.2 e-09). CI95 for linear fit are shaded in gray. (B) Correlation of observed and expected ratio of PfDHFR-TS_glmS and PfFC_glmS genomic DNA (Pearson’s r = 0.9939; P = 5.6e-06). CI95 for linear fit are shaded in gray. [file peerj-07-6713-s003.png]

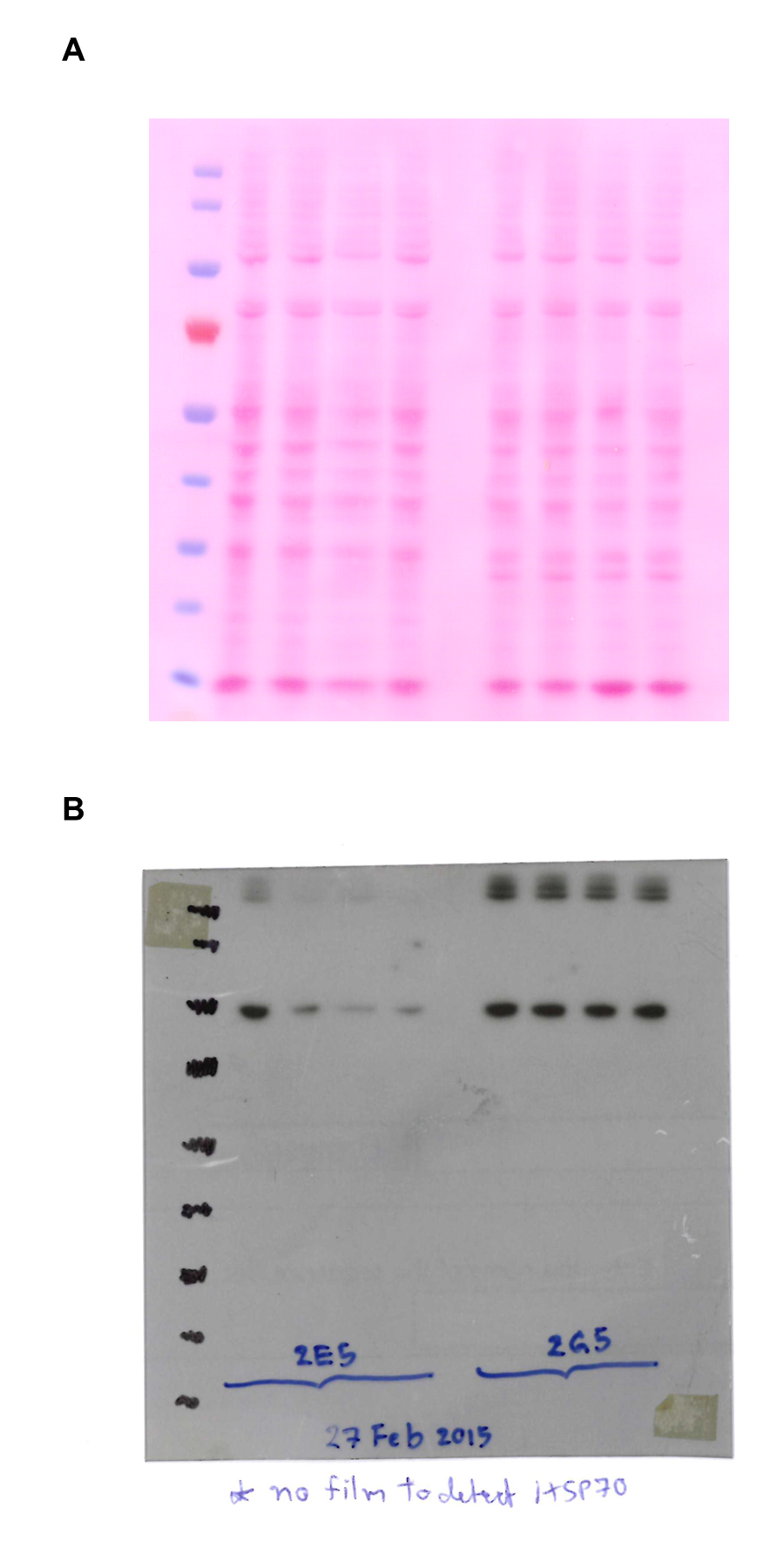

Supplement: Supplemental Information 4 — (A) Uncropped image of Ponceau-S stained membrane (cropped image in Fig. 2A, top panel). (B) Uncropped image of anti-GFP detection (cropped image in Fig. 2A, bottom panel). [file peerj-07-6713-s004.png]

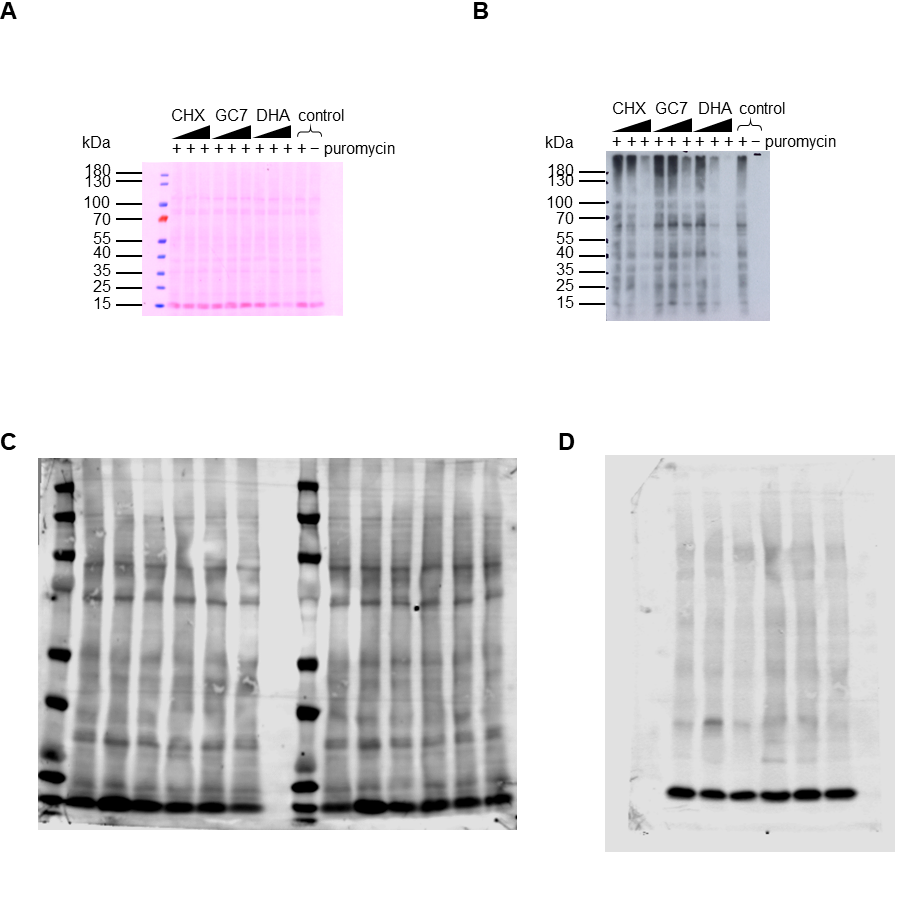

Supplement: Supplemental Information 5 — Parts (A–B): Validation of the puromycilation assay. Parasites were pre-treated with growth-inhibitory drugs for 1 h prior to puromycin exposure (five µM for 10 min). Proteins were extracted from harvested parasites and separated by electrophoresis. (A) Total protein stained with Ponceau-S. The migrations of PageRuler Plus Prestained Protein ladder (Thermo Scientific) standards are indicated on the left. (B) Puromycilated nascent peptides were detected with anti-puromycin antibody.The migrations of PageRuler Plus Prestained Protein ladder (Thermo Scientific) standards are indicated on the left. Increasing doses of drugs are indicated by the wedges above the lanes: cycloheximide (CHX, doses 1, 10, 100 µM); dihydroartemisinin (DHA, doses 0.01, 0.10, 1 µM) and N1-guanyl-1,7-diaminoheptane (GC7, doses 50, 100, 1,000 µM). Control lanes of samples from parasites not treated with growth-inhibitory compound are marked “control.” The specificity of the anti-puromycin antibody was tested by a control parasite culture in which parasites were not exposed to puromycin, marked “−puromycin.” (C) Uncropped image of PVDF membrane stained with REVERT (cropped image in Fig. 3A). (D) Uncropped image of hypusinated proteins detected with anti-hypusine antibody (cropped image in Fig. 3B). [file peerj-07-6713-s005.png]

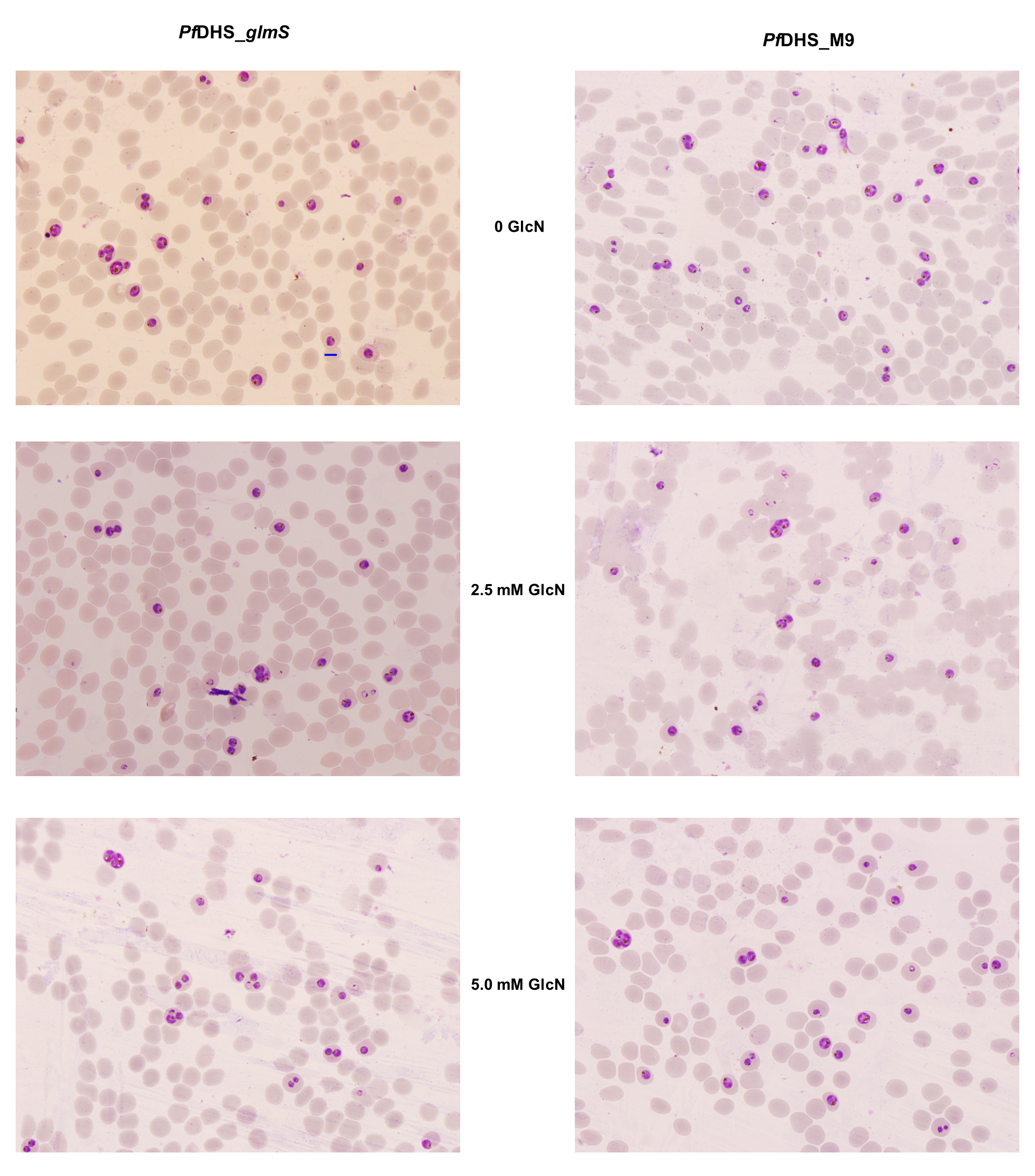

Supplement: Supplemental Information 6 — Ring-stage synchronized transgenic parasites PfDHS_glmS and PfDHS_M9 were treated with 0, 2.5 or 5.0 mM glucosamine (GlcN) for 72 h prior to hypusination and puromycilation assay. Thin film specimens were stained with Giemsa and images captured from 100× oil immersion microscopy. The scale bar (blue line) in the top-left image (PfDHS_glmS; 0 mM GlcN) represents five µm. [file peerj-07-6713-s006.png]

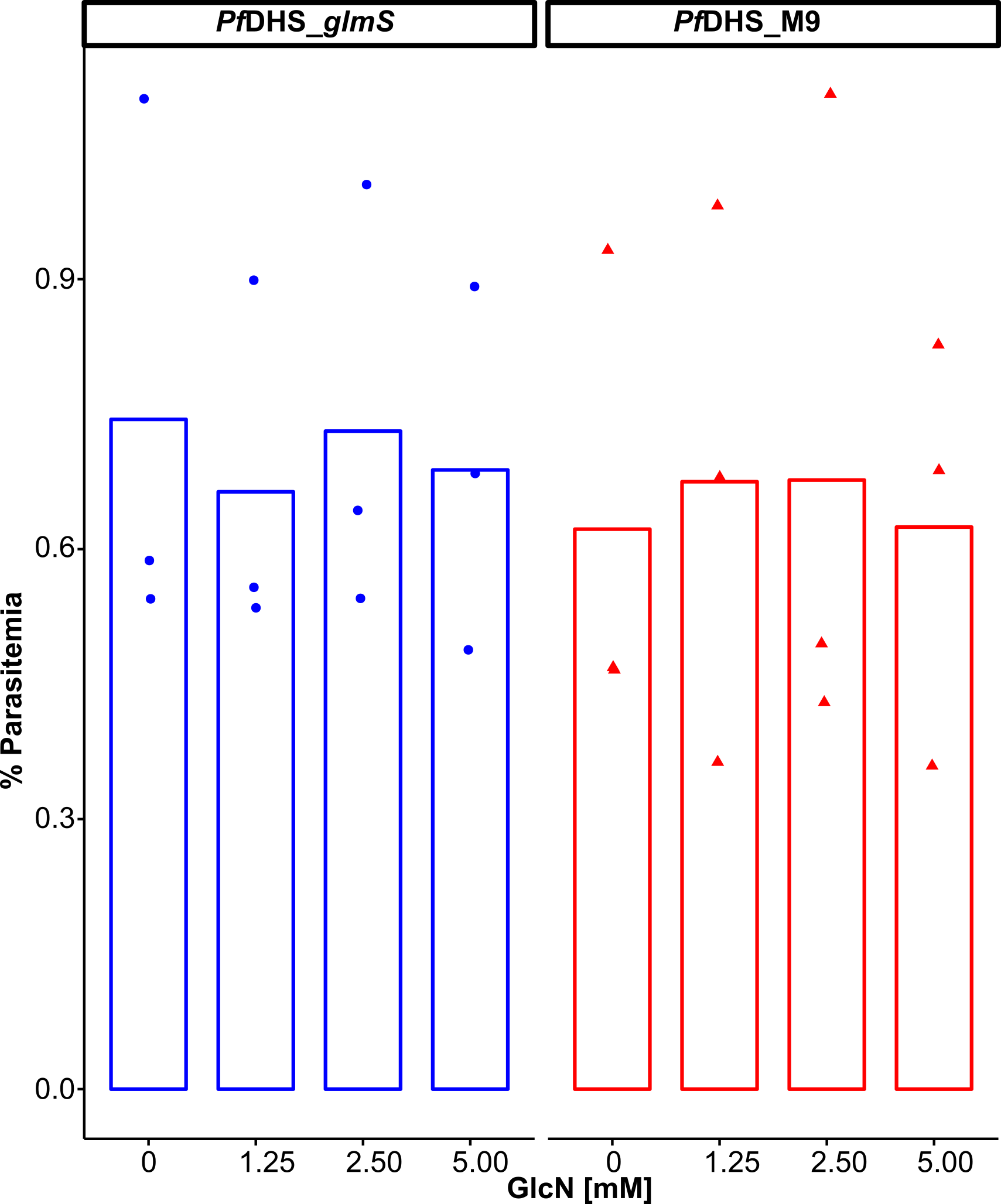

Supplement: Supplemental Information 7 — Ring-stage synchronized transgenic parasites PfDHS_glmS and PfDHS_M9 were cultured separately and diluted to 0.1% parasitemia. The cultures were treated with 0, 1.25, 2.50, or 5.00 mM glucosamine (GlcN) for 72 h. Parasite counts at 72 h were determined from thin film specimens stained with Giemsa. Counts were determined from at least 10,000 red blood cells per specimen. The data shown are from three independent experiments; mean values are shown as bars. [file peerj-07-6713-s007.png]

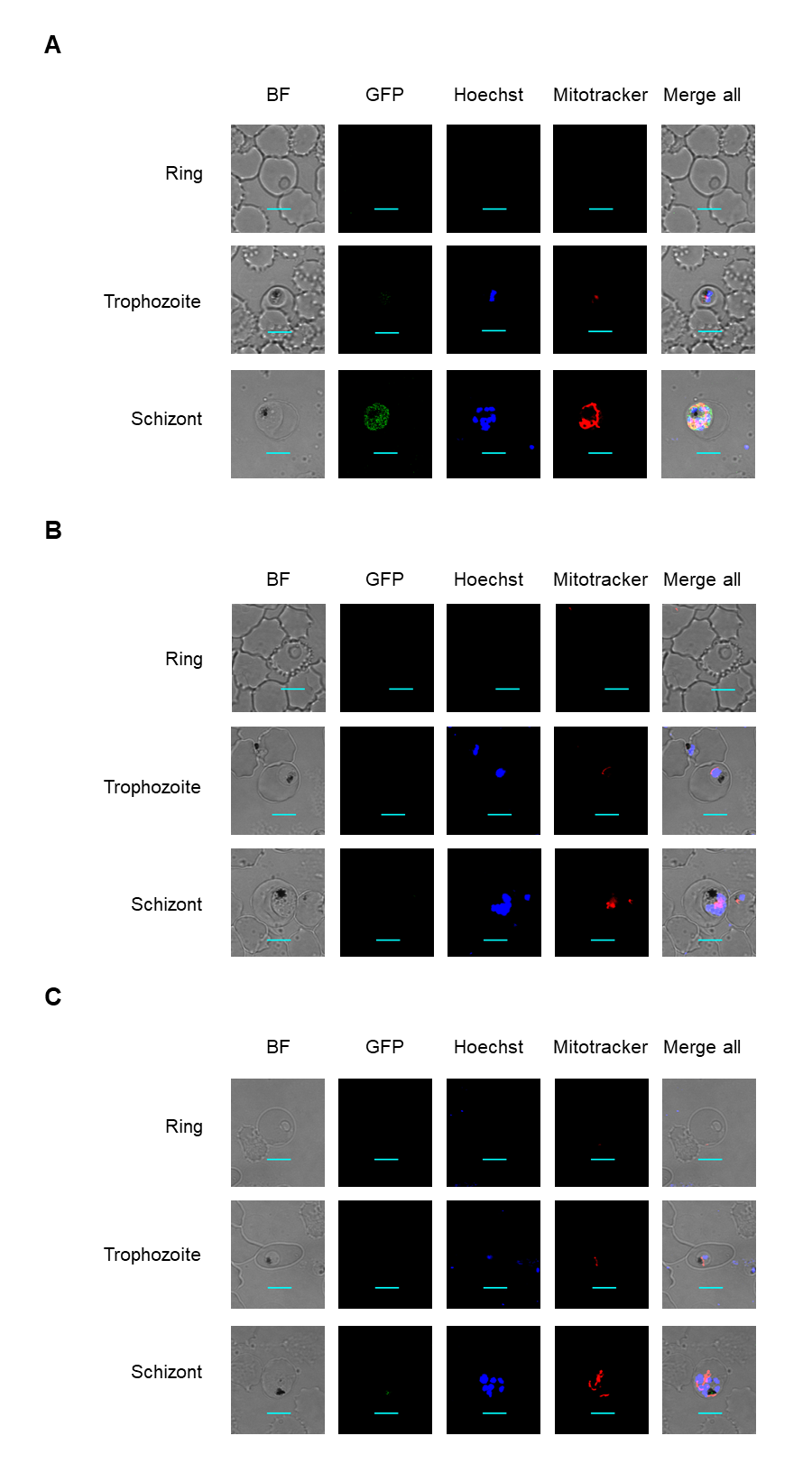

Supplement: Supplemental Information 8 — Representative confocal microscopic images of PfDHFR-TS_glmS (A), parental 3D7 (B) and PfFC_glmS (C) parasites at ring, trophozoite and schizont stages. Parasite nuclei were stained with Hoechst 33342. Mitochondria were stained with Mitotracker. Composite images from merging Hoechst, GFP, and Mitotracker fluorescence signals with the Bright-field (BF) image are shown in the panels on the far-right. Scale bars = 5 µm. [file peerj-07-6713-s008.png]

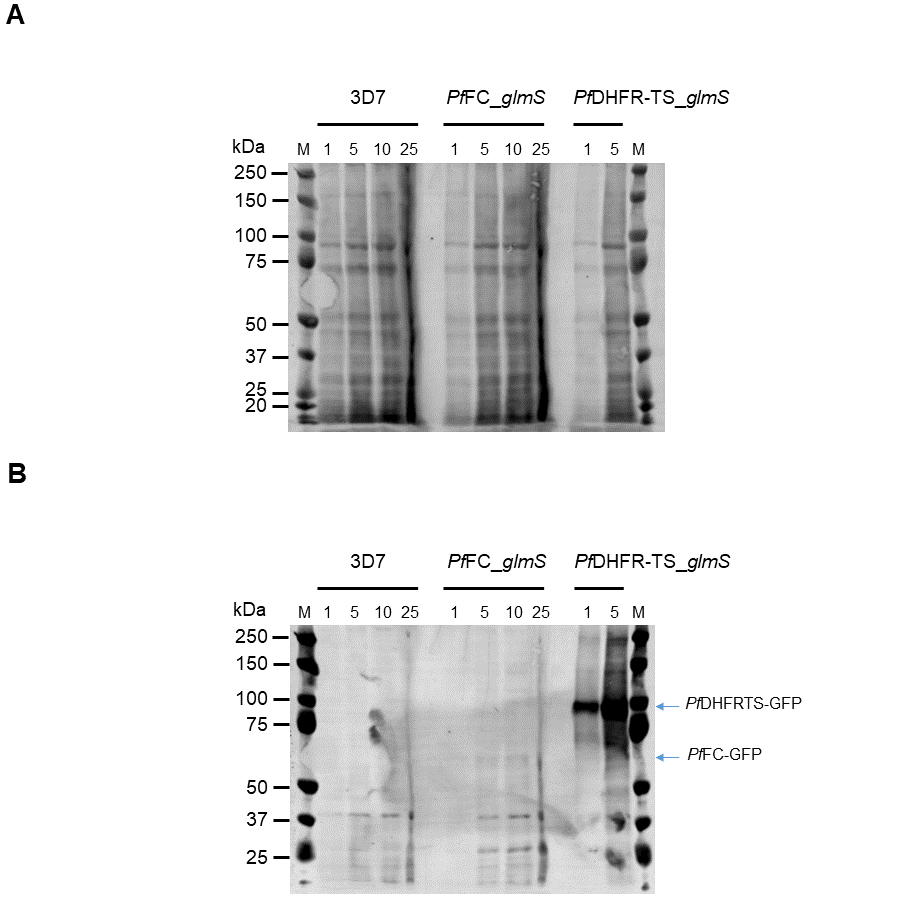

Supplement: Supplemental Information 9 — Protein extracts were obtained from 3D7, PfFC_glmS, and PfDHFR-TS_glmS parasites. The amount of extract loaded in each lane equivalent to the numbers of parasites is indicated above each lane (1, 5, 10, and 25 million). Proteins were separated in 4–12% NuPAGE gel and transferred to PVDF membrane. (A) Total protein stained with REVERT. (B) GFP-tagged proteins detected with anti-GFP antibody. Lanes marked “M” indicate PageRuler Plus Prestained Protein ladder (Thermo Scientific). The migrations Protein ladder standards are indicated on the left. The migrations of bands expected for PfFC-GFP (68 kDa) and PfDHFRTS-GFP (98 kDa) are indicated by arrows on the right. [file peerj-07-6713-s009.png]

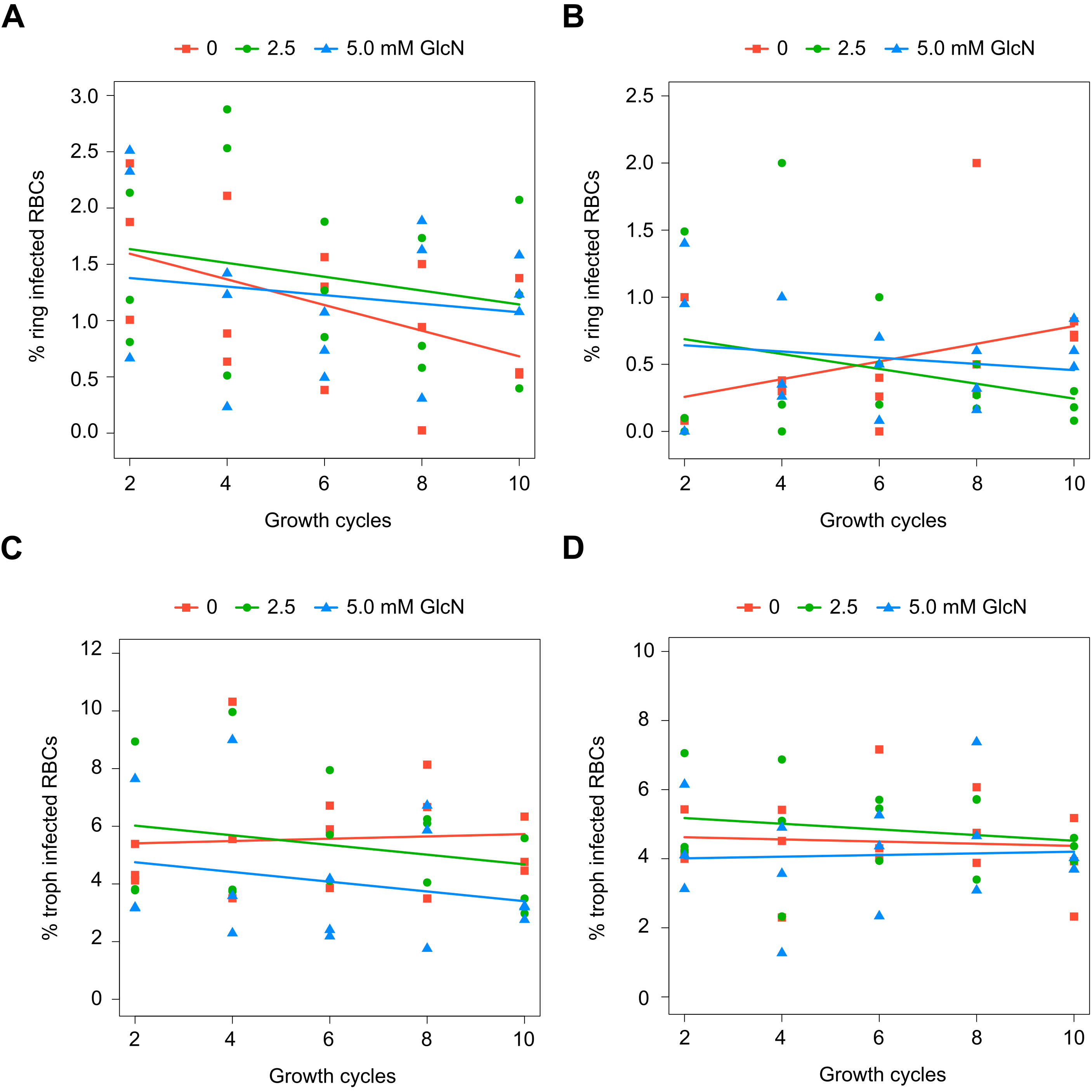

Supplement: Supplemental Information 10 — Synchronized cultures of parental 3D7 (A, C) and PfFC_glmS (B, D) control parasites were established with treatment of 0, 2.5, and 5.0 mM glucosamine (GlcN). The cultures were diluted to approximately 0.1% total parasitemia every two growth cycles. Infected red blood cells were enumerated from Giemsa-stained thin smears. The percentages of ring (A, B) and trophozoite (C, D) parasite-infected red blood cells were taken as growth values for modeling. The data from three independent experiments for each condition are shown. The lines on the graphs are the linear mixed effect models of growths at the indicated treatment doses of GlcN. P-values from likelihood ratio test: 0.92 (3D7, rings); 0.81 (3D7 trophs); 0.33 (PfFC_glmS rings); 0.89 (PfFC_glmS trophs). [file peerj-07-6713-s010.png]

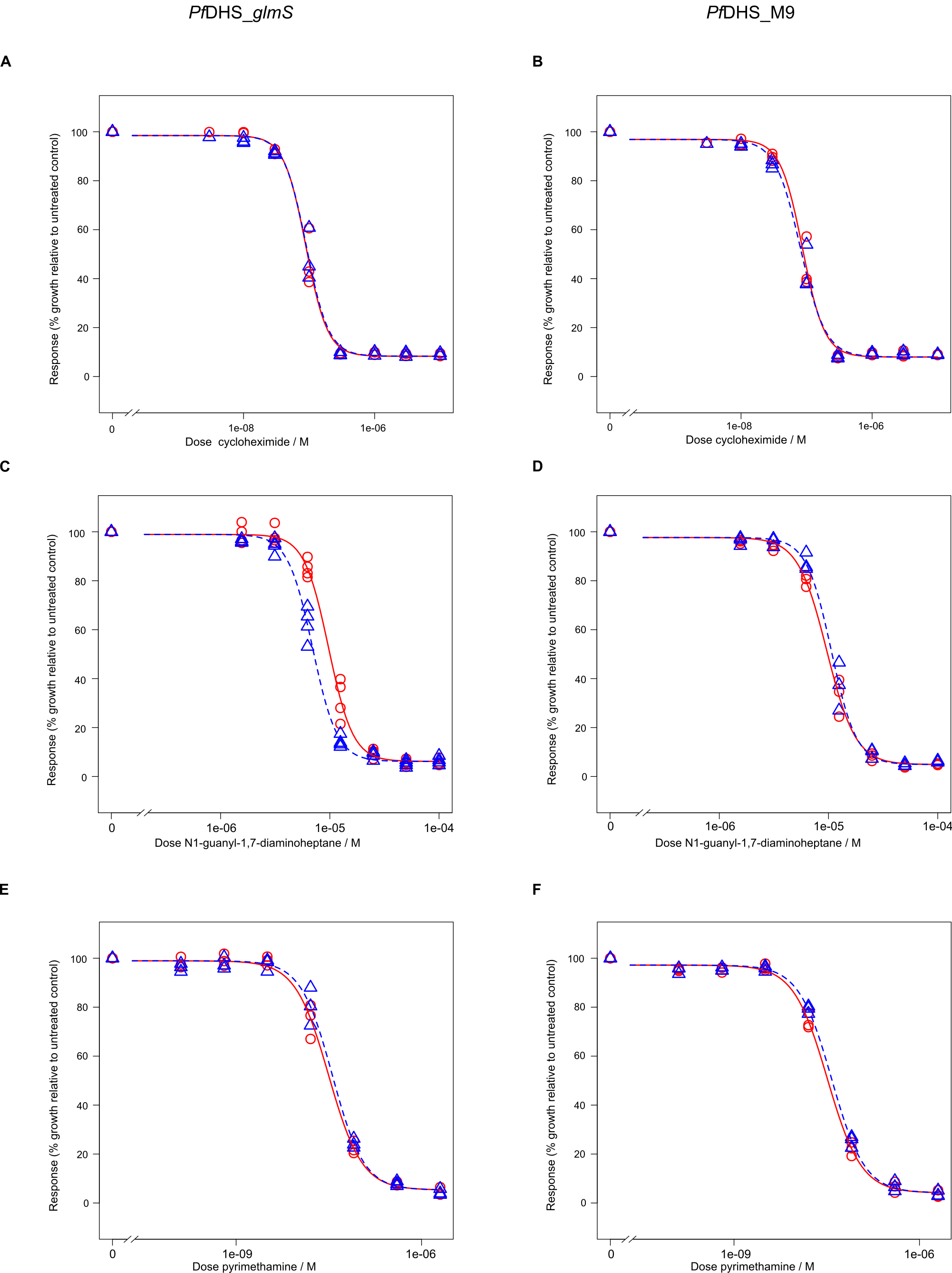

Supplement: Supplemental Information 11 — Data are shown from dose-response assays of growth-inhibitory compounds tested against transgenic parasites PfDHS_glmS (A, C, and E) and PfDHS_M9 (B, D, and F). The data for assays with 2.5 mM glucosamine (GlcN) co-treatment are shown as blue triangles and data without GlcN co-treatment are shown as red circles. The curves are the fitted models from the log-logistic dose-response equation with top and bottom shared between with/without GlcN co-treatment. Growth inhibitory compounds tested: cycloheximide (A, B); N1-guanyl-1,7-diaminoheptane (C, D) and pyrimethamine (E, F). [file peerj-07-6713-s011.png]

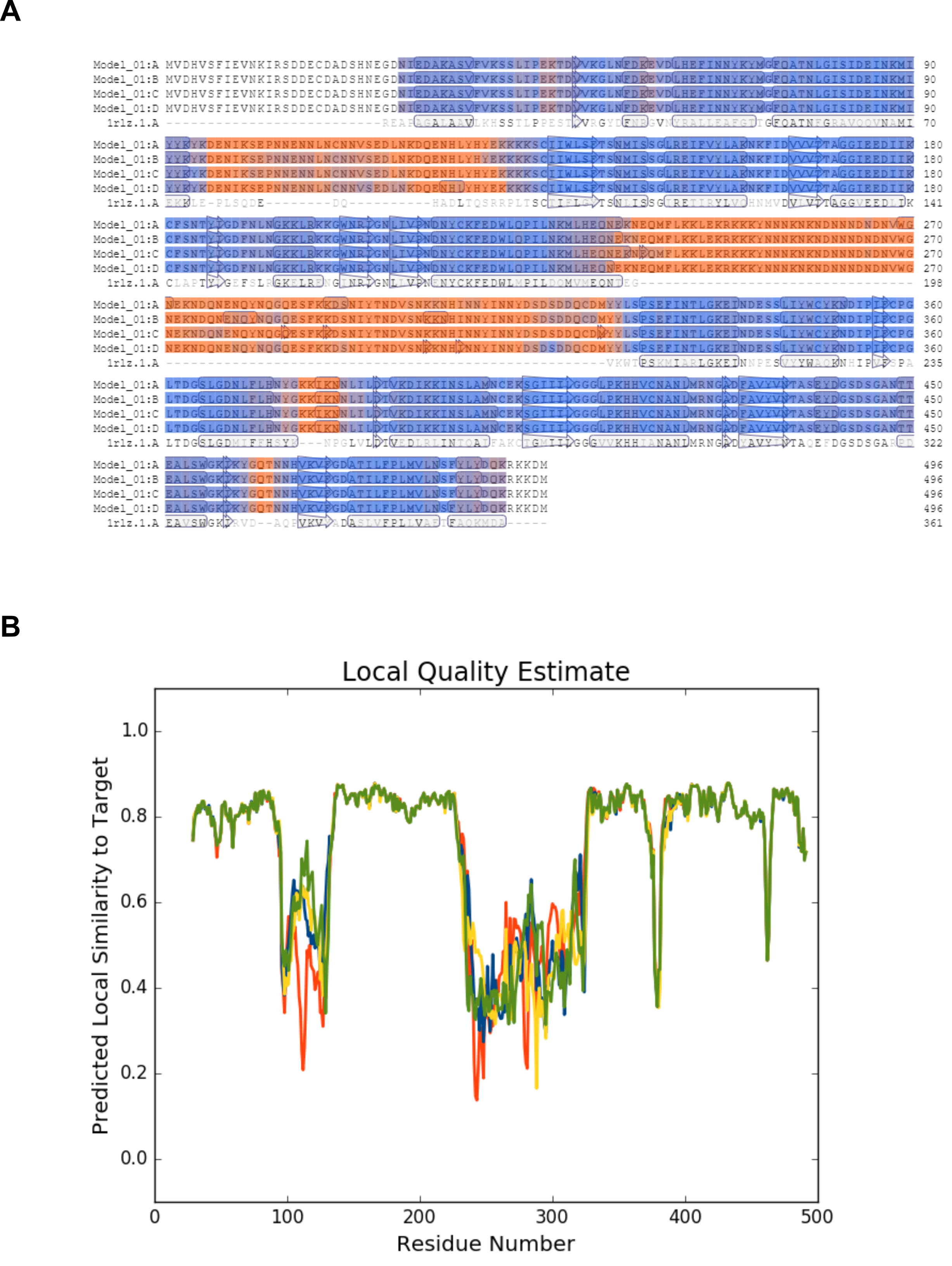

Supplement: Supplemental Information 12 — (A) Alignment of human DHS (PDB: 1RLZ) with PfDHS generated by the SWISS-MODEL server. Secondary structure elements are indicated on the protein residues (oblong, alpha helix; arrow, beta-sheet). (B) Graph of local quality estimate for the homology model structure of PfDHS. Q-MEAN score (y-axis) is plotted against PfDHS residue number. Subunits of a putative tetramer are plotted on the same axes (red, subunit A; blue, subunit B; yellow, subunit C; green, subunit D). [file peerj-07-6713-s012.png]

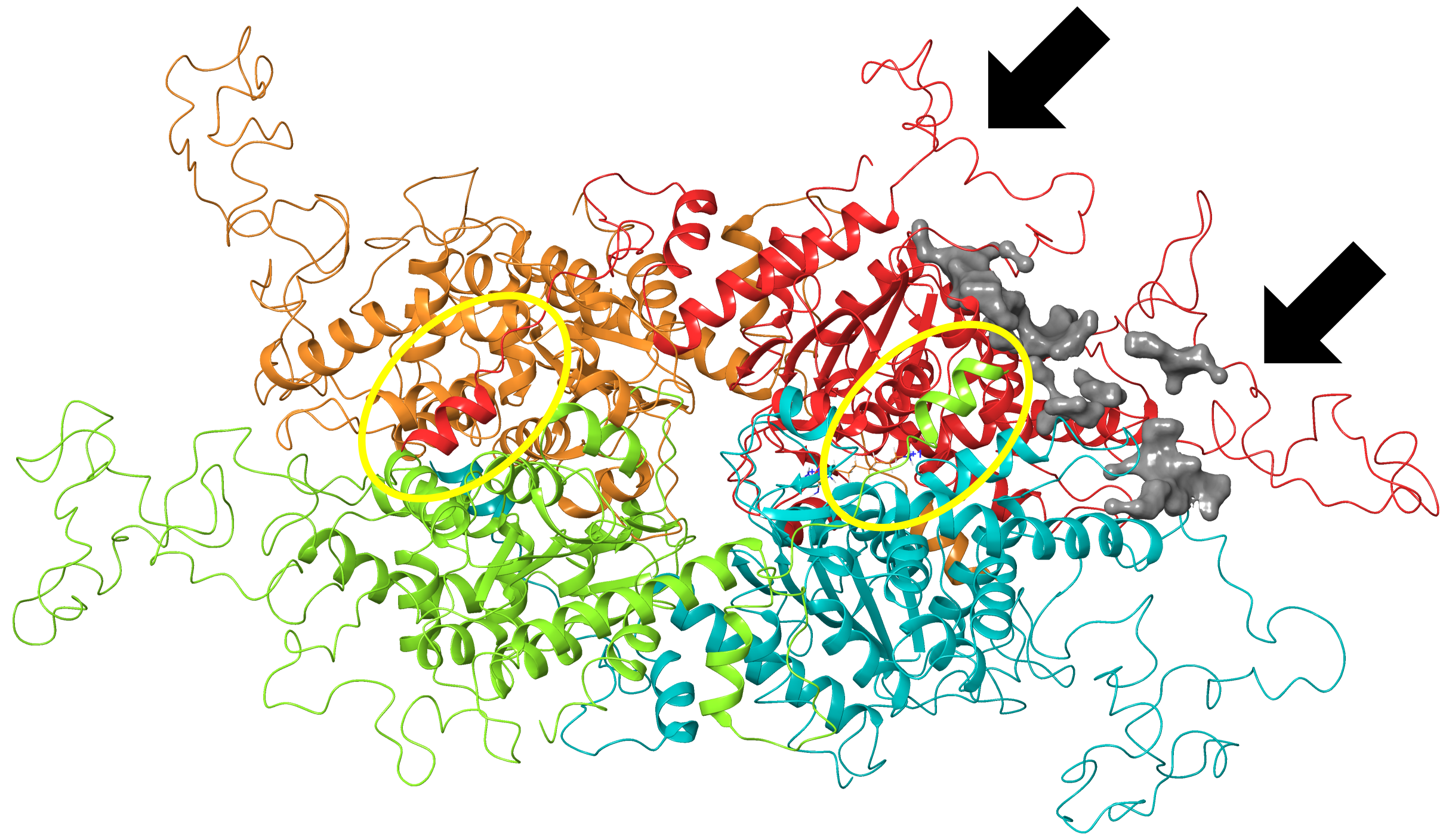

Supplement: Supplemental Information 13 — The second top-scoring ligand binding site in PfDHS identified by SiteMap is shown in gray. This site is located in-between PfDHS inserts Asp96–Lys131 and Glu235–Lys323 (indicated by arrows) and putative ball-and-chain motifs outlined in yellow. [file peerj-07-6713-s013.png]
